# Palliative care at any stage of amyotrophic lateral sclerosis: a prospective feasibility study

**DOI:** 10.3389/fmed.2023.1204816

**Published:** 2023-09-13

**Authors:** Jocelyn Zwicker, Ian C. Smith, Jill Rice, Rebekah Murphy, Ari Breiner, Susan McNeely, Maria Duff, Usha Buenger, Belinda Zehrt, Danica Nogo, Christine L. Watt

**Affiliations:** ^1^Faculty of Medicine, University of Ottawa, Ottawa, ON, Canada; ^2^Department of Medicine, The Ottawa Hospital, Ottawa, ON, Canada; ^3^Ottawa Hospital Research Institute, Ottawa, ON, Canada; ^4^Bruyère Research Institute, Ottawa, ON, Canada; ^5^Bruyère Continuing Care, Ottawa, ON, Canada; ^6^Queensway Carleton Hospital, Ottawa, ON, Canada; ^7^The Rehabilitation Centre, Ottawa, ON, Canada

**Keywords:** palliative care, anxiety, depression, quality of life, amyotrophic lateral scelerosis, virtual consultations

## Abstract

**Introduction:**

Many patients with amyotrophic lateral sclerosis (ALS) receive palliative care (PC) very late or not at all. The impact of PC on patients with ALS and caregivers has not been quantified. Study goals included (1) measuring the impact of early PC on quality of life and mood of patients/caregivers and (2) describing patient/caregiver satisfaction with PC.

**Methods:**

The study was a non-randomized, prospective feasibility study of patients with ALS being treated at The Ottawa Hospital ALS Clinic and their caregivers. Exclusion criteria were age < 18 years, inability to complete questionnaires, and prior receipt of PC. The ALS Specific Quality of Life-Revised (ALSSQOL-R) questionnaire (patients only) and Hospital Anxiety and Depression Scale (HADS) were completed at regular intervals for up to 2 years. Patients accepting a PC consultation completed a post-PC satisfaction survey. Primary outcome measures included ALSSQOL-R and HADS scores compared before and after PC consultation, and between groups receiving and not receiving a PC consultation. Secondary outcome measures included responses on the post-PC satisfaction survey (1 = strongly disagree, 5 = strongly agree).

**Results:**

39 patients with ALS (age 66 ± 10 years, median time from diagnosis = 6 months) and 22 caregivers were enrolled. 32 patients had a PC consultation (30 were virtual). Patients and caregivers agreed with statements that the PC consult was helpful (mean ± SD = 4.54 ± 0.60, range = 3–5) and they would recommend PC to others with ALS (4.59 ± 0.59, range = 3–5). Participants disagreed with statements that the consult would have been better later in disease course (1.87 ± 0.80, range = 1–4) and that it took too much time/energy (1.44 ± 0.85, range = 1–4). Average ALSSQOL-R scores worsened significantly over time. HADS and ALSSQOL-R scores did not significantly differ between groups receiving and not receiving PC.

**Conclusion:**

Patients with ALS and their caregivers found virtual PC consultations beneficial irrespective of disease duration or severity. Offering routine PC to all patients with ALS is feasible and should be considered as part of standard care.

**Clinical trial registration:**

https://clinicaltrials.gov/ct2/show/NCT04257760, identifier NCT04257760.

## Introduction

Amyotrophic lateral sclerosis (ALS) is an incurable, neurodegenerative condition characterised by progressive motor weakness, dysphagia, dysarthria, respiratory insufficiency and, within a median of 2–5 years, death. The disability associated with ALS results in many biopsychosocial needs and impacts patient and caregiver spiritual and emotional wellbeing (1). Given the complex needs of patients with ALS, palliative care (PC) involvement intuitively makes sense and has been recommended by professional organizations (2). PC may be provided to varying degrees by the members of the ALS care team however there are no evidence-based guidelines for initiating specialist PC for patients with ALS (3, 4). Currently, less than half of ALS patients see a PC specialist, even at the end of life (5, 6).

PC specialists have expertise in advance care planning, goals of care discussions, management of complex symptoms and providing psychological support both for families and their loved ones. Different models of care have been proposed, including offering specialist PC to all patients with ALS or offering PC only to patients who fulfill certain criteria (7, 8). Given the limited number of PC physicians, offering PC to patients with ALS at any stage of illness may not be feasible. There are also concerns that PC may not be accepted by all patients with ALS as previous studies have reported that ALS patients and caregivers may have negative impressions of PC (9). If PC is not recommended for all patients with ALS, it is important to identify the patients who would benefit most.

The objectives of the study were to (1) determine the feasibility and acceptability of PC consultation (PCC) for all patients with ALS irrespective of functional impairment or time from diagnosis, (2) measure patient and caregiver satisfaction with PCC, and the impact on mood and quality of life, and (3) to identify which patients and caregivers are most likely to benefit from PCC.

We hypothesized that PCC would be feasible and beneficial for all patients with ALS and their caregivers at any stage of disease or functional impairment.

## Materials and methods

### Standard protocol approvals, registrations, and patient consents

Ethical approval for this study was granted by the Ottawa Health Science Network Research Ethics Board, protocol number 20200141-01H. The study was registered at ClinicalTrials.gov (NCT04257760). Verbal or written informed consent was obtained from all participants in the study.

### Study design and study participant recruitment

The study was a non-randomized, prospective study of patients with ALS and their caregivers who were treated at The Ottawa Hospital ALS Clinic. The Ottawa ALS Clinic is a multidisciplinary clinic that follows 100–115 patients longitudinally, including 40–60 new referrals per year. Sample size was determined by the number of individuals who met eligibility criteria and agreed to enroll between October 2020 and April 2022.

Patients were included if they were at least 18 years of age and were being followed at the ALS clinic. Patients with a clinical diagnosis of ALS, primary lateral sclerosis or progressive muscular atrophy were included. Caregivers of eligible patients were eligible to participate. Patients and caregivers were excluded if they were younger than 18 years, were cognitively impaired to the extent they could not reliably complete questionnaires, had received PC prior to the initiation of the study or were not able to understand English or French well enough to complete questionnaires. Most participants were invited to participate by a member of their circle of care however some participants self-referred after attending a virtual ALS Society meeting. COVID restrictions prohibiting in-person clinic or research visits were instituted shortly before recruitment to the study was initiated. As patients were being followed by the health care team over the phone or through video visits, the planned recruitment of patients in the ALS clinic was impossible. The protocol was therefore amended to permit consent of patients over the phone by study personnel and to include consultations using virtual visit software.

After a short virtual meeting with a PC specialist to explain the possible role of PC in their care, patient participants were asked if they wanted to receive a PCC. Caregivers were not offered a PCC if the patient declined a PCC but were able to participate in the PCC at the discretion of the patient. PCCs were performed by one of 3 board-certified PC specialists (CW, JR, and RM). Due to COVID restrictions PCCs were initially only offered virtually however as COVID restrictions were lifted in-person consultations were also offered. All questionnaires were conducted online or delivered by mail, depending on participant preference.

### Outcome measures

All study participants were asked to complete a brief questionnaire including relevant demographic information. The severity of ALS was measured using the revised Amyotrophic Lateral Sclerosis Functional Rating Scale (ALSFRS-R) which was completed by the PC physician with patients during the PCC. The ALSFRS-R measures 12 aspects of physical function including ability to swallow, breathe, perform activities of daily living, and walk. Each function is scored from 4 (normal) to 0 (no ability), and the maximum and minimum possible ALSFRS-R scores are 48 and 0, respectively (10).

The primary outcome measures were mood, measured using the Hospital Anxiety and Depression Scale (HADS), and quality of life, measured by the Amyotrophic Lateral Sclerosis Specific Quality of Life Revised Scale (ALSSQOL-R). The HADS is a self-assessment questionnaire which has been validated for outpatients (11). There are 7 questions assessing depression (HADS-D) and 7 questions assessing anxiety (HADS-A). Each question is scored from 0 to 3 with 3 denoting the highest level of anxiety or depression. Scores of 8 or higher on the depression or anxiety subscale are sensitive and specific for detecting cases (11). HADS scores of 0–7, 8–10, 11–17, and 18–21 indicate normal, mildly elevated, moderately elevated, and severely elevated feelings of depression or anxiety (11, 12).

The ALSSQOL-R is a validated measure of quality of life for patients with ALS consisting of 50 items, each rated between 0 and 10. The item scores are used to calculate an average QOL score and 6 domain scores (13). ALSSQOL-R scores of 10 are considered optimal for subdomains of Negative Emotion, Interaction with People and the Environment, Physical Symptoms, Intimacy, and Bulbar Function. Scores less than 10 indicate that there are problems which detract from quality of life. A score of 10 on the Religiosity subdomain indicates that the participant identifies strongly with a religion and engages more in religious practice. Study participants with ALS were asked to complete the HADS and the ALSSQOL-R at baseline, 1 month, 3 months and every 3 months for the entirety of the study period (up to 2 years). Study participants who were caregivers for patients with ALS were asked to complete the HADS at the same time intervals.

To help inform future clinical trials, sample size calculations were performed using the observed changes in HADS and ALSSQOL-R scores.

The secondary measure of patient and caregiver satisfaction with PCC was measured through the Satisfaction with PCC Questionnaire that we developed for the study. Patients and caregiver participants who met with a PC specialist were asked to complete this questionnaire after the initial PCC.

After the PCC each physician documented the consultation using a template created for the study. The consultations were individualized and performed according to standard clinical practice rather than any fixed study criteria. The qualitative analysis of the PCCs will be reported separately.

Process measures included the proportion of study candidates who consented to participate in the study, the proportion of participants who elected to have a PCC, rates of completion of questionnaires, the duration of the PCC, and the time from consent to the PCC.

Balancing measures included patient or caregiver reports of stress or fatigue with PCC as measured by the Satisfaction with PCC Questionnaire.

### Statistical analysis

Study subgroups (i.e., patients with PCC, patients without PCC, and caregivers) were compared directly using either Kruskal-Wallis ANOVA tests (HADS scores) or Mann–Whitney U tests (ALSSQOL-R scores). Changes in scores over time were assessed using Wilcoxon matched pairs signed rank tests.

Multivariate analysis was undertaken to identify participant characteristics that were associated with the highest patient or caregiver benefit from PCC. To this end, two correlation matrices were generated, one for caregivers and another for patients with ALS. Variables were correlated using Spearman’s rho, a non-parametric test. Variables included in the multivariate analysis for caregivers included weeks since their loved one was diagnosed with ALS, the caregiver’s baseline HADS-D and HADS-A scores, whether or not a PCC took place, PCC duration, the caregiver’s PCC satisfaction survey responses, and the ALSFRS-R score of the associated patient at the time of the initial PCC. Variables included in the multivariate analysis for patients included weeks since diagnosis with ALS, baseline ALSSQOL-R domain scores, baseline HADS-D and HADS-A scores, whether or not the participant received a PCC, PCC duration, ALSFRS-R total and single question scores at the time of the initial PCC, and PCC satisfaction survey responses. PCC satisfaction survey responses, ALSSFRS-R scores and duration of PCC are only available when a PCC took place. Significance was taken at α = 0.05.

## Results

### Demographic and clinical patient characteristics

Patient and caregiver demographics are shown in Table 1.

**Table 1:** Participant demographics.

Participant category	Patients	Caregivers
Age at study intake (years)	Mean: 66	
	Median: 68	
	SD: 10	
	Range: 45–91	
Sex (*n*)	M: 23	M: 5
	F: 13	F: 12
Time since patient’s diagnosis (months)	Mean: 26	Mean: 31
	Median: 6	Median: 5
	SD: 60	SD: 84
	Range: 1–348	Range: 1–348
Time since patient’s first ALS clinic visit (months)	Mean: 22	Mean: 26
	Median: 4	Median: 3
	SD: 18	SD: 83
	Range: 0–348	Range: 1–348
Education completed by patient or by caregiver (*n*)	< High school: 2	< High school: 0
	High school: 14	High school: 5
	College/university: 9	College/university: 8
	Advanced degree: 11	Advanced degree: 2
		No answer: 2
Marital status of patient or of caregiver (*n*)	Married/common law: 25	Married/common law: 17-
	Widowed: 4	Widowed: 0
	Divorced/separated: 5	Divorced/separated: 0
	Never married: 2	Never married: 0
Employment status of patient or of caregiver (*n*)	Full time: 2	Full time: 3
	Part time: 1	Part time: 0
	Not employed: 33	Not employed: 12
		No answer: 2
ALSFRS-R score at initial PCC	Mean: 36.4	
	Median: 38	
	SD: 8.2	
	Range: 12–48	

**Figure 1:**
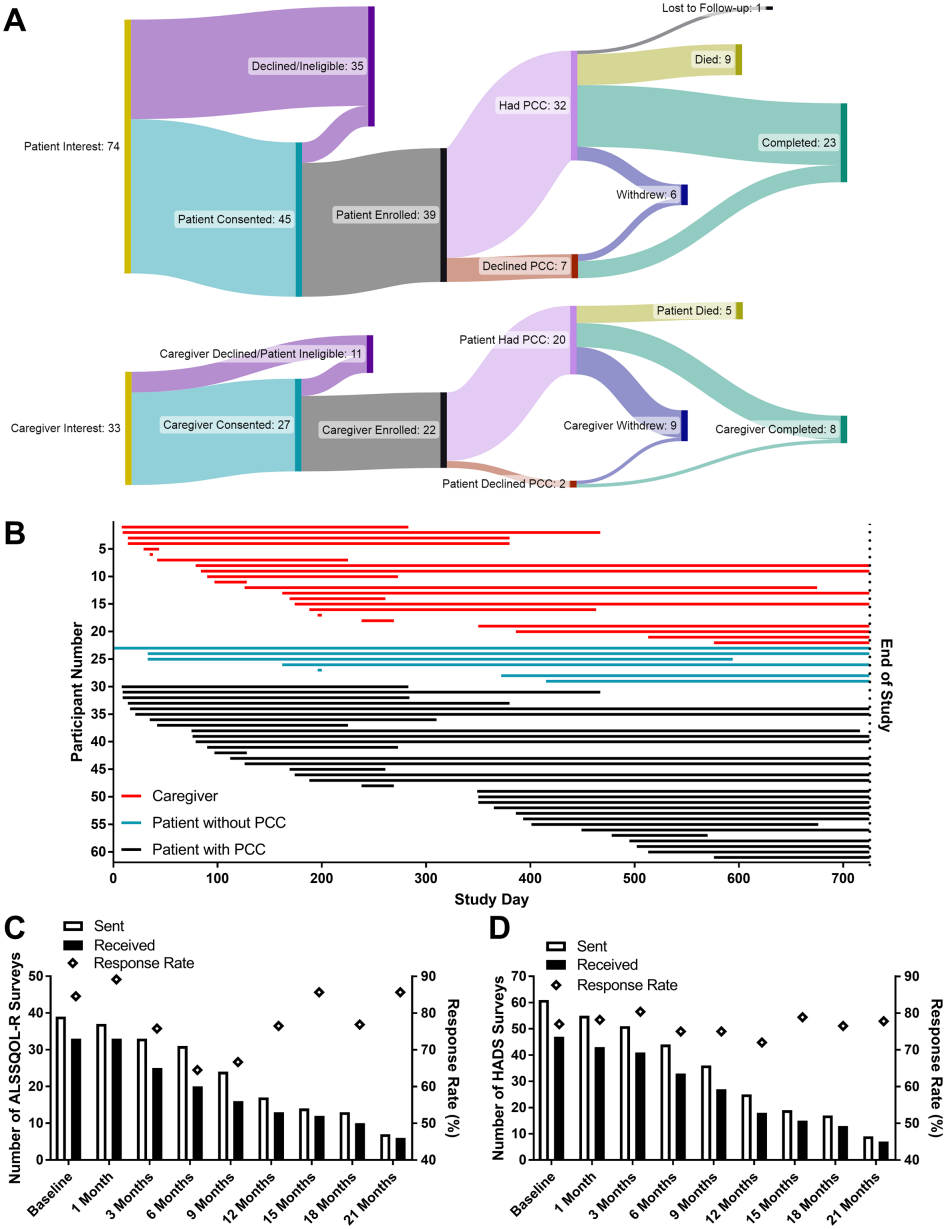
Enrollment and survey correspondence measures. The flow of patients and caregivers through the study **(A)**. Participants actively enrolled on the date the study closed are stated to have completed the study. The active enrollment period for all participants **(B)** beginning on either the date of the initial PCC or the date of the introductory PC phone call if a PCC was not desired. “End of Study” corresponds to the pre-planned study close date (October 31, 2022). The numbers of ALSSQOL-R **(C)** and HADS **(D)** surveys sent and received, as well as the percentage of completed surveys returned to researchers. Lower numbers of surveys sent at later time points reflect both participant attrition and the study design (featuring rolling enrollment with a fixed end date).

Depictions of patients and caregiver enrollment and flow through the study are shown in Figures 1A,B. 107 patients and caregivers were approached by study personnel. Documented reasons that eligible patients and caregivers withdrew from, or were removed from, the study following the consent process included death of the patient (*n* = 14), burdensome physical limitations (*n* = 3), patient dementia (*n* = 2), patient anxiety (*n* = 2), loss to follow-up (*n* = 1), a rapid transition to community PC (*n* = 1), and withdrawal of consent without specific explanation (*n* = 12).

### Process measures

A total of 640 surveys were distributed, and 502 were returned to research staff (78.4% response rate). Out of 33 paper surveys distributed to patients and caregivers by mail or during clinical visits, 17 (52% response rate) were returned to research staff. 607 surveys were sent electronically, and 485 received responses, including 2 which were completed by telephone conversation (79.9% response rate). Of the 62 intake surveys sent, 52 were completed and returned to research staff (85%). Of the 47 Satisfaction with PCC surveys sent, 38 were completed and returned to research staff (81%). Response rates for HADS (mean = 77.0%) and ALSSQOL-R (mean = 78.1%) are shown in Figures 1C,D, respectively. At 6 months post enrollment 19% of participants had withdrawn from the study.

### Uptake of palliative care consults

26 of 36 patients and 14 of 17 caregivers expressed interest in receiving a PCC in the intake survey and a further 4 patients and 1 caregiver expressed interest in receiving a PCC after the introductory meeting with a PC physician. Reason(s) given in the intake surveys for wanting or not wanting a PCC are summarized in Table 2. The most common reasons for wanting a PCC were information seeking (*n* = 17) and future planning (*n* = 13). The participants who were not interested in a PCC consultation indicated that either they had adequate supports or they were not ready “yet.” Though caregivers were asked about their desire for a PCC in the intake surveys, the final decision to have/not have a PCC was made by the patients after the introductory call with a PC doctor.

**Table 2:** Reasons given for wanting or not wanting a palliative care consultation.

Desire for PCC	Stated reasoning in intake survey (themed)	Patients (*n* mentioning)	Caregivers (*n* mentioning)
No (Not yet)	Not required yet.	3	1
No (Not yet)	Problems aren’t bad enough yet.	3	0
No (Not yet)	Not ready to discuss yet.	3	1
No	Existing supports are adequate.	1	1
Yes	Interested but vague on detail / left blank	5	7
Yes	Information seeking.	12	5
Yes	Deferral to spouse.	0	1
Yes	Need help now.	3	2
Yes	Future planning/End of life arrangements.	11	2
Yes	Altruism.	1	0
Yes	No reason not to.	1	0

All 32 initial PCCs and 44 follow-up visits were performed in the 2 years of the study. All were virtual video consultations with the exception of 1 consultation which occurred in person and 1 which was conducted over the telephone due to technical difficulties. On average the time between consenting to the study and the PCC was 33 ± 30 days for patients (median: 23 days, range: 9–130 days). Initial consults lasted for an average of 69 ± 17 min (median = 70 min, range = 30–105 min). This depended on study doctor, with PCCs lasting, 92 ± 11 min, 73 ± 11 min, and 59 ± 17 min when performed by Doctor 1, Doctor 2, and Doctor 3, respectively (Doctor 1 > Doctor 2 > Doctor 3; all *p* < 0.05). Throughout the study 2 PC physicians working 1 day a week on this project were able to perform the necessary initial and follow-up PCCs.

**Figure 2:**
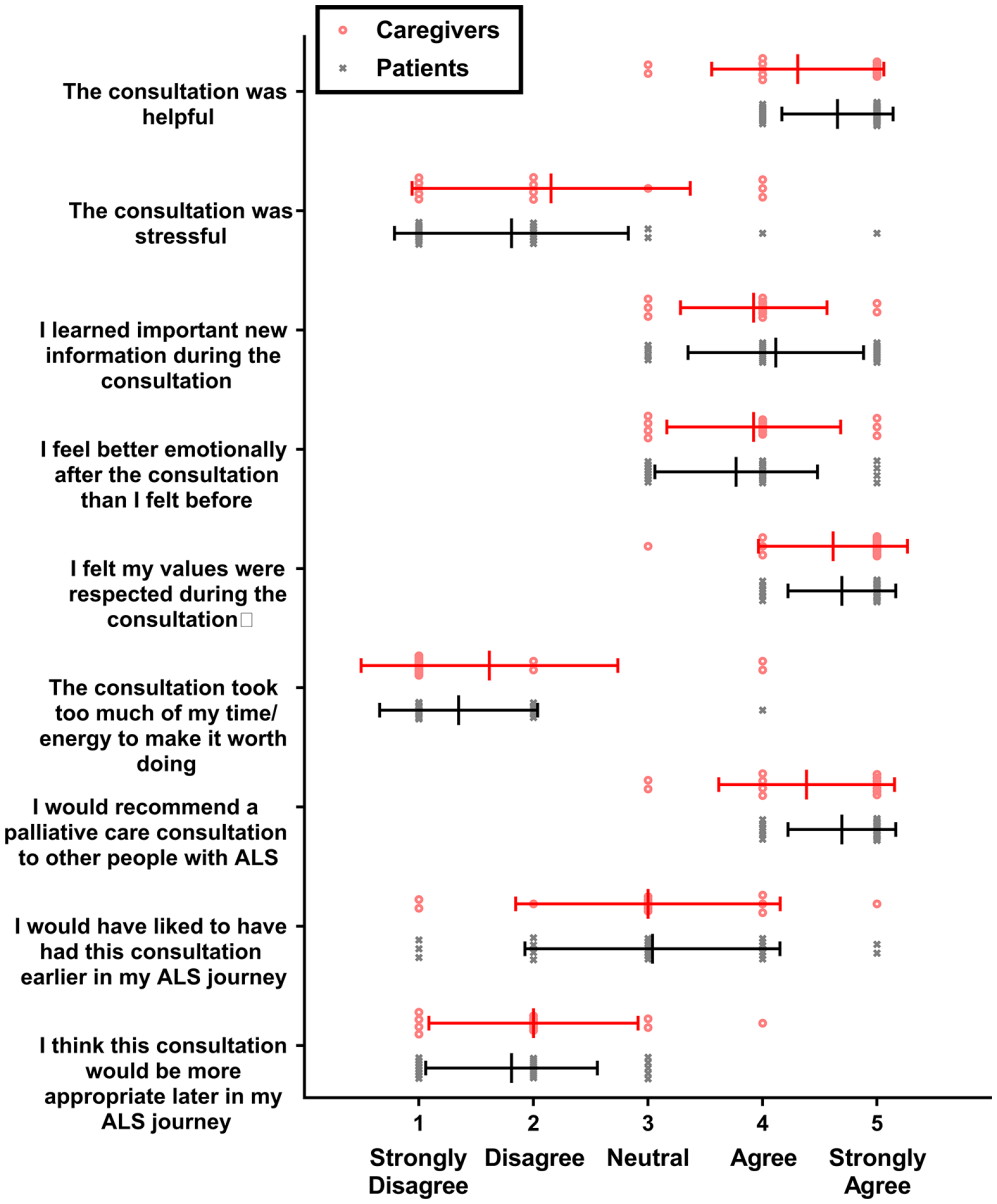
Satisfaction with PCC survey scores. Individual values are shown for caregivers (red circles; *n* = 13) and patients with ALS (gray X’s; *n* = 26), along with means and standard deviations (lines and bars).

Patients and caregivers were asked to complete a satisfaction with PCC survey following the first PCC. Answers to satisfaction with PCC surveys are summarized in Figure 2. Overall patients and caregivers rated the PCCs favorably. Disagreement with the statement that the patient would have liked the PCC to come earlier in the ALS journey should be interpreted as the PCC coming at the right time for the survey taker because no patient and only one caregiver agreed with the statement that the PCC would be more appropriate later in the ALS journey.

On average, participants did not feel the PCC was stressful or required too much energy to make it worth doing. Satisfaction with PCC survey responses were compared between study doctors; no statistically significant differences were found between doctors when examining patients alone, caregivers alone, or patients and caregivers together (data not shown).

### Quality of life and mood outcomes

**Figure 3:**
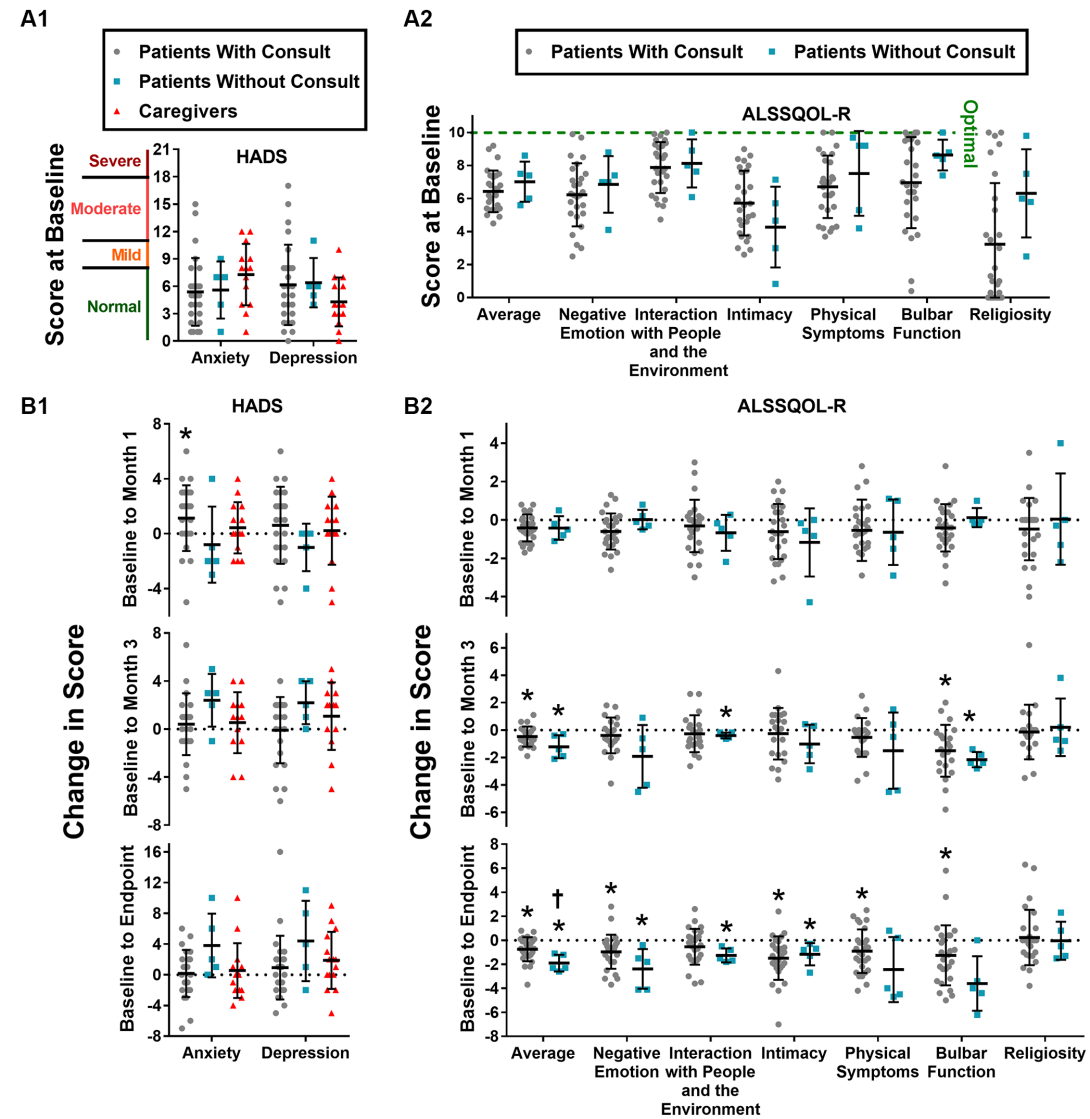
HADS and ALSSQOL-R scores. HADS-A and HADS-D scores at intake **(A1)** and changes in score from baseline to month 1, to month 3, and to final completed survey **(A2)**. Average and domain scores of the ALSSQOL-R survey at intake **(B1)** and changes in scores from baseline to month 1, month 3, and to final completed survey **(B2)**. Individual scores, group means, and standard deviation are shown. Increases in HADS scores correspond to worsening mood. With exception of religiosity, decreases in ALSSQOL-R scores correspond to worsening quality of life. High religiosity scores reflect high importance of, and involvement in, religious practices. * *p* < 0.05 vs. score at intake. † *p* < 0.05 vs. Patients with Consult.

Mood was measured using HADS scores, and results are summarized in Figure 3. HADS-D scores at intake were moderately elevated in 5 patients and 0 caregivers, mildly elevated in 5 patients and 1 caregiver, and normal in 21 patients and 13 caregivers. HADS-A scores at intake were moderately elevated in 3 patients and 3 caregivers, mildly elevated in 4 patients and 3 caregivers, and normal in 24 patients and 6 caregivers. No significant differences in HADS-D or HADS-A scores were detected between patients with PCC, patients without PCC, and caregivers at intake. At one month, HADS-A scores increased significantly (*p* < 0.05) by 1.13 ± 2.81 HADS units among patients who received a PCC but were not elevated from baseline at 3 months or any subsequent time point. No statistically significant changes in HADS-A scores were detected among patients that did not receive a PCC or among caregivers.

Quality of life was measured using the ALSSQOL-R, and results are summarized in Figure 3. On average, patients without PCC scored higher than patients with PCC on 6 of the 7 ALSSQOL-R domains at baseline, but none of these differences reached the level of statistical significance (*p* > 0.05). ALSSQOL-R Average scores were significantly lower at 1-month, 3-month, and last survey time points than at baseline among the patients with PCC. Patients without PCC also had significantly (*p* < 0.05) lower ALSSQOL-R Average scores at 3-months and at last-survey time points than at baseline. Compared to baseline, patients with PCC also reported significant declines in ALSSQOL-R domains of Negative Emotion (at 1 month and last-survey), Intimacy (at last-survey), Physical Symptoms (at last-survey), and Bulbar Function (at 3-months and last-survey). Patients without PCC reported significant (*p* < 0.05) declines in the ALSSQOL-R domains of Interactions with People and the Environment (at 3-months and last-survey), Intimacy (at last-survey), and Bulbar Function (at 3-months).

In a *post hoc* analysis comparing the first and last completed surveys, ALSSQOL-R Average score fell significantly more (*p* < 0.05) in the patients without PCC (from 7.0 ± 1.2 to 5.1 ± 1.2 ALSSQOL-R units; a decline of 1.9 ± 0.7 ALSSQOL-R units) than the patients with PCC (from 6.4 ± 1.3 to 5.7 ± 1.4 ALSSQOL-R units, a decline of 0.7 ± 1.0 ALSSQOL-R units). Patients without PCC reported increases in HADS-D and HADS-A scores of 4.4 and 3.8 points, respectively, but these increases did not reach the threshold of statistical significance (*p* = 0.13 and *p* = 0.11 for HADS-D and HADS-A, respectively). No other significant differences in the magnitude of change in ALSSQOL-R or HADS scores were detected between patients who received a PCC and patients who did not.

### Multivariate analysis

#### Caregivers

**Figure 4:**
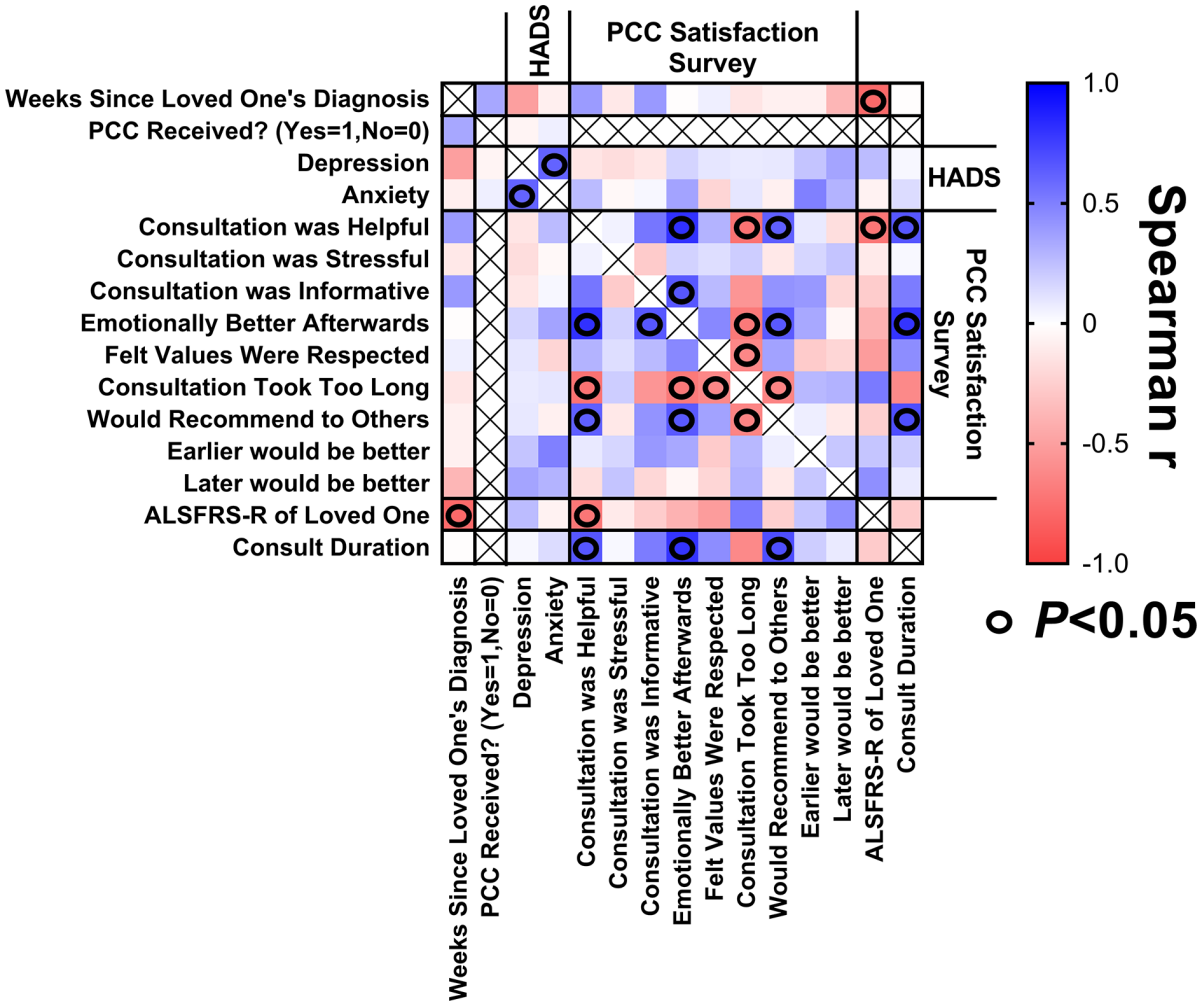
Correlation matrix for caregivers. Correlations between (1) weeks since loved one’s diagnosis at enrollment, (2) caregiver’s baseline HADS scores, (3) satisfaction with PCC survey responses, (4) ALSFRS-R score of loved one at time of PCC, and (5) PCC duration.

A correlation matrix was generated to explore relationships between study variables relating to caregivers of patients with ALS (Figure 4), in hopes of identifying characteristics of caregivers reporting the greatest benefit from PCC. The PCC duration was positively and significantly correlated with caregiver reports that the PCC was helpful, the participants felt emotionally better afterwards, and the participants would recommend a PCC to others. A low (worse) patient ALSFRS-R score was associated with caregiver reports that the PCC was helpful. There were no statistically significant correlations between weeks since patient diagnosis or high caregiver HADS-A or HADS-D scores with respect to caregiver satisfaction with PCC.

#### Patients

**Figure 5:**
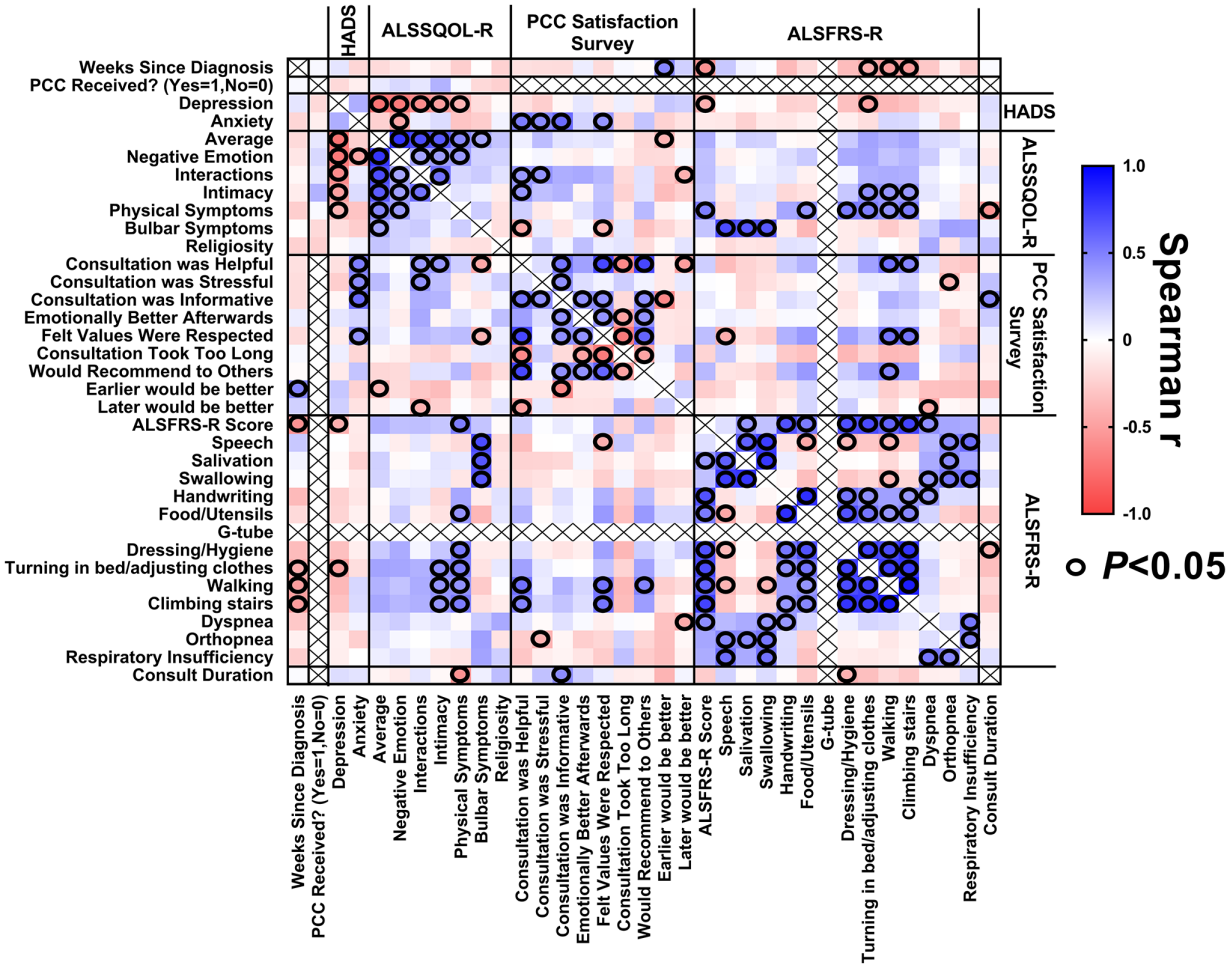
Correlation matrix for patients. Correlations between (1) weeks since diagnosis at enrollment, (2) baseline HADS scores, (3) baseline ALSSQOL-R scores, (4) satisfaction with PCC survey results, (5) ALSFRS-R scores at the time of PCC, and (6) PCC duration.

A correlation matrix was generated to explore the relationship between study variables relating to patients with ALS (Figure 5) to identify characteristics of patients reporting the greatest benefit from PCCs. A longer duration PCC was associated with patient reports that the PCC was informative. Patients with a longer duration of illness were more likely to report they would have like to have the PCC earlier in their ALS journey.

Patients with a lower (worse) average ALSSQOL-R score were more likely to report that they would have preferred that the PCC happen earlier in their ALS journey. Patients with higher domain scores on Interactions with People and the Environment were more likely to find the PCC helpful but also stressful. Patients with higher Intimacy domain scores were associated with reporting the PCC was helpful. Patients with more bulbar symptoms were also more likely to report the PCC was helpful and that their values were respected.

The ALSFRS-R score did not correlate significantly with patient satisfaction with PCC.

Higher (worse) HADS-A scores at baseline correlated with PC consultation being stressful, informative, helpful, and participants feeling that their values were respected.

There was internal consistency as longer duration of ALS corresponded to a lower ALSFRS-R score. Higher HADS-D scores correlated to lower scores on ALSSQOL-R overall and on most subcategories. Low bulbar scores on the ALSSQOL-R correlated with low scores for speaking, swallowing and salivation on the ALSFRS-R.

## Discussion

This study demonstrates that patients with ALS and their caregivers find PCC beneficial irrespective of the time since diagnosis or the degree of functional impairment. Previous studies have described the topics covered by PC specialists when counselling patients with ALS (7, 8, 14). The most frequent foci of discussion include advanced care planning including code status, goals of care including personal values, and symptom management such as treatment of pain and muscle spasms. This study describes the clinical impact of PC consultations. We report patient and caregiver perceptions of those interactions in a semi-quantitative fashion. We also provide data on the quantitative impact on mood using the HADS scores. This is the same tool that was used to demonstrate the effectiveness of early PC in oncology patients (15). Finally, we provide quantitative data on the impact on quality of life which is the ultimate target of PCCs.

Among these measurements the most compelling was the degree of patient and caregiver satisfaction. All patients reported that the PCC was helpful, and none would have preferred it later in the disease course. On average, patients and caregivers disagreed with the statements that PCC took too much time to be worth doing or that it was stressful. PCCs were most highly rated by patients with high levels of anxiety and worse bulbar function, and by caregivers of patients with low function, as determined by ALSFRS-R scores. There was a statistically significant increase in patient anxiety within 1 month of the PCC, but patient anxiety returned to baseline after 3 months. As the magnitude of increase in anxiety score was 0.30 SD of mean HADS-A score at 1 month, it failed to meet the 0.50 SD threshold established for a minimal clinically important difference of health-related quality of life questionnaires for chronic diseases (16). Although the satisfaction questionnaire is a subjective, unvalidated scale it reflects measurable, meaningful feedback from patients endorsing an intervention with no evidence of harm. Given the results of this study, we recommend offering a PCC to all patients with ALS.

Despite previous reports that patients or caregivers have negative feelings about PC or misunderstand PC (9) the vast majority of patients agreeing to participate in this study accepted a PCC when it was offered. The rate of consent to PCC in this study (82%) was higher than reported in a previous study of universal referral (75%) (8). In part this may have been related to selection bias as only patients that enrolled in the study were offered an early PCC. In addition, insurance authorization was not an obstacle. The introduction to PC provided by a PC specialist prior to the PCC was also a factor as 4 patients changed their mind and accepted the PCC after the short description. Finally, the PCCs were convenient for patients to attend as they were conducted virtually at a separate time from the multidisciplinary ALS clinic.

This study highlights the efficacy of virtual care in this patient population where physical limitations may limit in-person clinical care or trials. Virtual PCCs were well received by patients and caregivers. This provides flexibility for clinics where space is a premium, eliminates the travel time of in-person home visits by the PC specialist, and reduces the physical, monetary, and temporal demands on patients by eliminating the need to travel to a clinic. Most importantly, the increased flexibility allows for visits to occur separately from long, fatiguing in-person multidisciplinary ALS clinic visits. In other studies, fatigue and the length of the clinic visit are reasons ALS patients have cited for limiting the duration of the PCC (7) or choosing not to see a PC provider (8). The majority patients were able to complete questionnaires online, making virtual evaluation of PCC feasible. Other benefits of virtual evaluation included reduced need and cost of paper products, and better accommodating patients with severe weakness by avoiding writing utensils.

Despite the positive findings on the satisfaction with PCC questionnaire, we were unable to demonstrate a statistically significant improvement in mood or quality of life on validated questionnaires at the pre-specified time points. Over the course of the disease, mean HADS scores increased more in the group of patients who did not receive a PCC compared to patients who did but this was not statistically significant. Although a decline in quality of life is expected in progressive condition such as ALS, there was less of a decline in quality of life on the ALSSQOL-R from baseline to end of study participation in patients who received PCC compared to patients who did not but not at other time points. The lack of statistically significant differences in the ALSSQOL-R at other time points and in the HADS scores may in part be due to the small comparison group as most patients chose to receive a PCC, and lower-than-expected enrollment due to COVID measures.

Currently specialist PCCs for patients with ALS are not part of standard of care and many ALS multidisciplinary clinics do not include a PC physician. In one study of North American ALS multidisciplinary clinics, less than a third of sites included a PC specialist and patients were referred to the PC specialists at an advanced stage of ALS (5). In Ontario, Canada, less than half of patients receive a PCC in the last year of life (6). The American Academy of Neurology has been criticized for dropping PC from its evidence-based ALS recommendation because “measurable benefit from PC service in ALS had not been proven” (17). The present study provides evidence that patients with ALS and their caregivers find PC beneficial, which can be used to advocate for more outpatient services. As the minority of patients with ALS require regular follow-up appointments, and some patients transition to community PC or hospice care, it is possible to offer PCCs to 32 patients at an ALS clinic with a minimum of 1 PC physician working 2 days per week.

If further evidence of the effectiveness of PC for patients with ALS is desired, this study provides critical information for planning a randomized controlled trial. Demonstrating an improvement in HADS and ALSSQOL-R scores will require a larger sample size as the scores changed little over time. The lack of change of the quality of life of patients with progressive deterioration in health has been previously described in PC studies and is described as a “response shift,” or a realignment of patient’s standards and values in the context of the limitations imposed by the illness (18). In addition, the attrition rate of study participation (Figure 1) suggests that measurement of change would be best detected within the first 6 months. To help guide future randomized clinical trials, sample size calculations predict that PCC would have a statistically significant impact on mood and quality of life scores of ALS patients at 3-months post-intervention with group sizes of 27 (HADS-A), 23 (HADS-D), and 18 (ALSSQOL-R Average). A multi-center trial would be required to recruit the requisite number of patients particularly given the described enrollment rates and response rates.

### Strengths

This was a prospective study during which we performed multivariate analysis including several clinical features as well as quantitative outcomes at many time points. The rate of survey completion was similar or better compared to previous survey studies on the ALS population [reported survey response rates of 79.5% (19), 59.2% (20), and 44.4% (21)]. We included a semi-quantitative evaluation of patient and caregiver satisfaction with PCC. Patient participation was not limited based on insurance coverage (8). PCC and follow-up were performed according to clinical judgement, reflecting real world practice. PCCs were almost exclusively virtual which provides important insights into the effectiveness of that practice in this patient population.

### Limitations

The study population may be relatively homogeneous as the study recruited patients and caregivers from a single ALS multidisciplinary clinic. However, patient characteristics are similar to those reported previously with an average age at diagnosis of 64 years and an increased prevalence in males (1.77,1) (22). The study was conducted during the COVID pandemic and therefore the results may not be reflective of usual care. During this time there were many changes to the care patients with ALS were receiving including closure of the ALS multidisciplinary clinic for several months prior to study enrollment and subsequent opening with limited resources, various degrees of social isolation of participants, and limitations on homecare. Due to the low number of participants choosing to enroll in the study without a PCC, our ability to compare the intervention group to the non-intervention group is limited. Due to the low number of participants overall, our ability to detect small differences at certain time points (e.g., 6 or 12 months after diagnosis) for patients prior to receiving a PCC vs. after receiving a PCC was poor. This was a non-randomized study so there may have been a selection bias towards patients and/or caregivers who were more in need or appreciative of PC.

In summary, this study demonstrates that patients with ALS and their caregivers find virtual PCCs beneficial irrespective of functional impairment or time from diagnosis. Patients with high levels of anxiety and worse bulbar function, and caregivers of patients with low function may benefit the most, however offering virtual PCC to all patients with ALS is feasible. Offering routine PC should be considered for all patients with ALS. A large multi-centre trial is needed to better understand the impact of PC on quality of life in patients with ALS.

## Data availability statement

The anonymized data sets analysed during this study are available to qualified investigators upon reasonable request to the corresponding author.

## Ethics statement

The studies involving humans were approved by Ottawa Health Science Network Research Ethics Board. The studies were conducted in accordance with the local legislation and institutional requirements. The ethics committee/institutional review board waived the requirement of written informed consent for participation from the participants or the participants’ legal guardians/next of kin because the COVID-19 epidemic imposed limitations on patient contact. Participants were read a Verbal Consent Script by a research assistant and subsequently provided verbal consent to participate in this minimal-risk study (approved by the OHSN-REB; protocol #20200141-01H).

## Author contributions

JZ, AB, MD, SM, UB, and CW contributed to the design and/or conceptualization of the study. JZ, IS, JR, RM, MD, DN, SM, UB, BZ, and CW had a major role in the acquisition of data. JZ, IS, and CW contributed to the analysis and/or interpretation of the data. JZ, IS, JR, RM, AB, UB, BZ, and CW contributed to drafting and/or revising the manuscript for intellectual content. All authors contributed to the article and approved the submitted version.

## Funding

This project was supported by a grant from the ALS Society of Canada. IS was supported by the Eric Poulin Centre for Neuromuscular Disease fund.

## Conflict of interest

The authors declare that the research was conducted in the absence of any commercial or financial relationships that could be construed as a potential conflict of interest.

## Publisher’s note

All claims expressed in this article are solely those of the authors and do not necessarily represent those of their affiliated organizations, or those of the publisher, the editors and the reviewers. Any product that may be evaluated in this article, or claim that may be made by its manufacturer, is not guaranteed or endorsed by the publisher.

## Abbreviations

ALS: amyotrophic lateral sclerosis
ALSFRS-R: revised amyotrophic lateral sclerosis functional rating scale
ALSSQOL-R: amyotrophic lateral sclerosis specific quality of life-revised questionnaire
HADS: hospital anxiety and depression scale
HADS-A: hospital anxiety and depression scale-anxiety subsection
HADS-D: hospital anxiety and depression scale-depression subsection
PC: palliative care
PCC: palliative care consultation
SD: standard deviation
